# Identification of Bacterial Cell Wall Lyases via Pseudo Amino Acid Composition

**DOI:** 10.1155/2016/1654623

**Published:** 2016-06-29

**Authors:** Xin-Xin Chen, Hua Tang, Wen-Chao Li, Hao Wu, Wei Chen, Hui Ding, Hao Lin

**Affiliations:** ^1^Key Laboratory for Neuro-Information of Ministry of Education, Center of Bioinformatics and Center for Information in Biomedicine, School of Life Science and Technology, University of Electronic Science and Technology of China, Chengdu 610054, China; ^2^Department of Pathophysiology, Southwest Medical University, Luzhou 646000, China; ^3^School of Computer Science and Technology, Harbin Institute of Technology Shenzhen Graduate School, Shenzhen, Guangdong 518055, China; ^4^Department of Physics, School of Sciences, and Center for Genomics and Computational Biology, North China University of Science and Technology, Tangshan 063000, China

## Abstract

Owing to the abuse of antibiotics, drug resistance of pathogenic bacteria becomes more and more serious. Therefore, it is interesting to develop a more reasonable way to solve this issue. Because they can destroy the bacterial cell structure and then kill the infectious bacterium, the bacterial cell wall lyases are suitable candidates of antibacteria sources. Thus, it is urgent to develop an accurate and efficient computational method to predict the lyases. Based on the consideration, in this paper, a set of objective and rigorous data was collected by searching through the Universal Protein Resource (the UniProt database), whereafter a feature selection technique based on the analysis of variance (ANOVA) was used to acquire optimal feature subset. Finally, the support vector machine (SVM) was used to perform prediction. The jackknife cross-validated results showed that the optimal average accuracy of 84.82% was achieved with the sensitivity of 76.47% and the specificity of 93.16%. For the convenience of other scholars, we built a free online server called* Lypred*. We believe that* Lypred* will become a practical tool for the research of cell wall lyases and development of antimicrobial agents.

## 1. Introduction

Bacteria are widely distributed on the earth, a significant proportion of which can cause disease. The antibiotic can efficiently treat infectious diseases caused by pathogens. However, antibiotics abuse may cause bacterial drug resistance. Thus, there is an ever-increasing need to find new ways to address this important issue [1, 2]. In the search for more effective therapeutic strategies, great effort has been placed on the study and development of lyases, which benefits from high potency activity toward drug-resistant strains and a low inherent susceptibility to emergence of new resistance phenotypes [3–7].

In 1896, the British bacteriologist Hankin found that the bacteriophage has antibacterial activity [3]. Subsequently, in 1921, Brunoghe and Maisin used bacteriophage to treat staphylococcal skin disease in France, which was the first reported application of bacteriophage to treat infectious diseases [8]. Maxted [9], Krause [10], and Fischetti et al. [11] found that the lysates of Group C streptococci infected with C1 bacteriophage contain an enzyme which has the ability to lyse streptococci and their isolated cell walls. The enzyme is called endolysin which is encoded by bacteriophage gene. It can cause bacteria death by degrading cell wall. It has been reported that 10 ng endolysins can lead to 10^7^ bacteria's lysis within 30 seconds [4, 12].

Autolysins are another kind of lyases that are functionally similar to endolysins except they are bacteria-encoded enzymes [13]. It has been reported that autolysins play important roles in several fundamental biological phenomena, such as cell wall enlargement, genetic transformation, flagella extrusion, cell division, and lysis induced by fl-lactam antibiotics, as well as in the “suicidal tendencies” of pneumococci [14–16].

Due to their special biological activity, lyases have been applied in antibacteria drug development. Thus, it is necessary to perform intensive research on lyases to understand the antibacterial mechanism. Although wet experiments are an objective approach for accurately recognizing the lyases, they are often time-consuming and costly. Due to the convenience and high efficiency, computational methods have attracted more and more attention. Many algorithms such as common support vector machine (SVM) [17–19], structured SVM [20], artificial neural network (ANN) [21], Random Forest (RF) [22], *K*-nearest neighbor (KNN) [23–25], Bayesian classifier [26, 27], Mahalanobis discriminant [28, 29], LibD3C [30], genetic algorithm [31], imbalanced classifier [32], learning to rank [33], and ensemble learning [34, 35] have been developed for protein function prediction. Various sequence features descriptors such as amino acid composition [36, 37], pseudo amino acid composition (PseAAC) [38], physicochemical properties [39], secondary structure features [40], and N-peptide composition [41] were proposed to represent protein sequences [42].

To deal with the problem about lyases prediction, recently, a method was developed to identify cell wall enzymes by using PseAAC and Fisher discriminant [43]. A maximum overall accuracy of 80.4% was obtained with the sensitivity of 66.7% and the specificity of 88.6% [43]. However, further work is needed due to the following reasons. (i) The prediction quality can be further improved. (ii) No web server for the prediction method in [43] was provided, and hence its usage is quite limited, especially for the majority of experimental scientists.

The present study was devoted to development of a new predictor for identifying lyases. For this purpose, an objective and strict benchmark dataset was constructed for training and testing the proposed model in which protein sequences were formulated by using an improved PseAAC. For the convenience of other scholars, a free online server called* Lypred* (at http://lin.uestc.edu.cn/server/Lypred/) was established.

## 2. Material and Method

### 2.1. Benchmark Dataset

A high quality dataset is the key to building a robust and accurate predictor. The lyases in bacteria or bacteriophage were regarded as positive samples which were derived from the UniProt [44]. Negative samples, namely, the nonlyases, were also derived from bacteriophage and downloaded from the UniProt. In order to guarantee the reliability of the benchmark dataset, we optimized the data according to the following standards: firstly, the sequences whose protein was with annotations of “Inferred from homology” or “Predicted” were excluded; secondly, we removed the sequences which are the fragments of other proteins; thirdly, the protein sequences containing unknown residues, such as “B,” “J,” “O,” “U,” “X,” and “Z,” were eliminated; fourthly, to avoid overestimation of prediction model that resulted from the high sequence identity, the CD-HIT program [45] was adopted to eliminate redundant sequence by setting the cutoff of sequence identity to 40%. As a result, a total of 68 lyases and 307 nonlyases were obtained to form the final benchmark dataset.

### 2.2. Features Extraction

A sequence can be represented by two different forms: one is the sequential form and the other is the discrete form [46]. The most common and straightforward way to characterize a protein is to use all the residues in its sequence written as follows:

(1) $$\begin{array}{c} P=R_{1}R_{2}R_{3}R_{4},\ldots ,R_{L-1}R_{L}, \end{array}$$

where *R*_1_, *R*_2_, and *R*_*L*_ are the 1st, 2nd, and *L*th amino acid residue of protein *P*, respectively. Based on the information, a query protein can be predicted by the BLAST or FASTA program. The results are always good for the query sequence which has high similar sequences in benchmark dataset; however, it fails to work when the similar sequences for the query sequence are not found in the training dataset [47]. Therefore, the similarity-based method is not suitable for the case that no homologue was found in the benchmark dataset. The discrete form can overcome the shortcoming and is easy to be treated in statistical prediction. Thus, it has been widely used in protein and DNA formulation [48, 49]. The PseAAC is a typical discrete form that has been widely used for protein function prediction [46, 50, 51].

It is well known that the polypeptide chains fold to tertiary structures based on the physicochemical properties of residues. Thus, it is not enough to analyze the residue compositions of protein molecules. Hence, we proposed to represent protein samples by using an improved PseAAC which includes not only *g*-gap dipeptide composition, but also correlation of physicochemical property between two residues.

According to the concept of PseAAC, a protein *P* with the length of *L* can be formulated in a (400 + *nδ*) dimension space as given by

(2) $$\begin{array}{c} D=\left[\begin{array}{c} f_{1}\\ f_{2}\\ \vdots \\ f_{400}\\ f_{401}\\ \vdots \\ f_{400+n\delta } \end{array}\right], \end{array}$$

where

(3) $$\begin{array}{c} f_{i}=\left\{\begin{array}{cc} \varphi_{i}, & 1\leq i\leq 400\\ \varepsilon_{i}, & 400<x\leq 400+n\delta , \end{array}\right. \end{array}$$

where *φ*_*i*_ denotes the normalized occurrence frequency of the *i*th kind of *g*-gap dipeptide in protein *P* formulated as

(4) $$\begin{array}{c} \varphi_{i}=\frac{n_{i}^{g}}{\sum_{i=1}^{400}n_{i}^{g}}=\frac{n_{i}^{g}}{L-g-1}, \end{array}$$

where *n*_*i*_^*g*^ (*i* = 1,2,…, 400) denotes the number of the *i*th *g*-gap dipeptide in *P*.

*ε*_*i*_ in (3) is the *i*-tier sequence correlation factor calculated by the following formulas:

(5) $$\begin{array}{c} \varepsilon_{400+1}=\frac{1}{L-1}\overset{L-1}{\underset{t=1}{\sum }}\theta_{t,t+1}^{1}\\ \varepsilon_{400+2}=\frac{1}{L-1}\overset{L-1}{\underset{t=1}{\sum }}\theta_{t,t+1}^{2}\\ \vdots \\ \varepsilon_{400+n}=\frac{1}{L-1}\overset{L-1}{\underset{t=1}{\sum }}\theta_{t,t+1}^{n}\\ \varepsilon_{400+n+1}=\frac{1}{L-2}\overset{L-2}{\underset{t=1}{\sum }}\theta_{t,t+2}^{1}\\ \varepsilon_{400+n+2}=\frac{1}{L-2}\overset{L-2}{\underset{t=1}{\sum }}\theta_{t,t+2}^{2}\\ \vdots \\ \varepsilon_{400+n+n}=\frac{1}{L-2}\overset{L-2}{\underset{t=1}{\sum }}\theta_{t,t+2}^{n}\\ \vdots \\ \varepsilon_{400+n\delta }=\frac{1}{L-\delta }\overset{L-\delta }{\underset{t=1}{\sum }}\theta_{t,t+\delta }^{n}\\ \;\left(\delta <L\right). \end{array}$$

The correlation *θ*_*x*,*y*_^*n*^ of physicochemical property between two residues is given by

(6) $$\begin{array}{c} \theta_{x,y}^{n}=\rho^{n}\left(\;R_{x}\;\right)\rho^{n}\left(\;R_{y}\;\right), \end{array}$$

where *ρ* ^*n*^(*R*_*x*_) denotes the *n*th physicochemical value of amino acid residue *R*_*x*_. The value is obtained by

(7) $$\begin{array}{c} \rho^{n}\left(\;R_{x}\;\right)=\frac{\rho_{0}^{n}\left(R_{x}\right)-\sum_{k=1}^{20}\rho_{0}^{n}\left(R_{k}\right)/20}{\sqrt{\sum_{t=1}^{20}{\left(\rho_{0}^{n}\left(R_{t}\right)-\sum_{k=1}^{20}\rho_{0}^{n}\left(R_{k}\right)/20\right)}^{2}/20}}, \end{array}$$

where *ρ*_0_^*n*^(*R*_*x*_) is the *n*th physicochemical original value of amino acid *R*_*x*_.

Thus, each protein sample can be expressed by 400 + *nδ* kinds of features according to (2)–(7).

### 2.3. Feature Selection

Some features are noise or redundant information which will reduce the predictive performance of classification models. Thus, it is very important to develop a method to evaluate the contribution of every feature to the classification. Here, we used ANOVA [52] to rank features defined as

(8) Fi=∑j=12mj∑s=1mjfis,j/mj−∑j=12∑s=1mjfis,j/∑j=12mj2∑j=12∑s=1mjfis,j−∑s=1mjfis,j/mj2/∑j=12mj−2,

where *F*(*i*) represents the *F*-score of the *i*th feature type, *f*_*i*_(*s*, *j*) is the feature value of the *i*th feature type of the *s*th sample in the *j*th protein type, and *m*_*j*_ is the number of samples in the *j*th protein type. It is obvious that the larger the *F*(*i*) value, the better the discriminative capability the *i*th feature has.

In order to eliminate the redundant features, we firstly ranked all features according to their *F*-score from high to low. The first feature subset only contained the feature with the largest *F*-score; then, a new feature subset was generated when the feature with the second largest *F*-score was added. The process was repeated until all features were added. The SVM was used to evaluate the performance for each feature subset. The feature subset with the best performance is deemed the optimal feature subset which does not contain redundant features.

### 2.4. Support Vector Machine

The SVM is a linear-classifier-based supervised machine learning method, which has been successfully used in many bioinformatics fields [48–51, 53–57]. To attain the goal of classification, SVM utilizes the kernel function to deal with the nonlinear transformation, and thus linear inseparable can be converted to a linear problem in high-dimension Hilbert space. In this work, the software LIBSVM [58] was used to execute SVM.

### 2.5. Performance Standard

To provide a more intuitive and easier-to-understand method to evaluate the prediction performance, we used the following criteria: the sensitivity (Sn), the specificity (Sp), Mathew's correlation coefficient (MCC), the overall accuracy (OA), and the average accuracy (AA), which were defined as

(9) Sn=TPTP+FNSp=TNTN+FPMCC=TP×TN−FP×FNTP+FP×TN+FN×TP+FN×TN+FPOA=TP+TNTP+FN+TN+FPAA=12×TPTP+FN+TNTN+FP,

where TP is the number of lyases that were correctly predicted, FN denotes the number of lyases that were predicted as the nonlyases, TN is the number of nonlyases that were correctly predicted, and FP denotes the number of nonlyases that were predicted as the lyases.

In addition, we also chose the receiver operating characteristic curve (ROC curve) to measure the performance of the proposed model. ROC curve is a kind of comprehensive index that is drawn by using (1 − Sp) as the abscissa and Sn as the ordinate. Thus, it reveals the continuous variable of Sn and Sp. Generally, we only need to calculate the area under the ROC curve (auROC). The greater the auROC is, the better the discriminate capability the prediction model has is.

## 3. Results and Discussion

### 3.1. Forecasting Accuracy

In this work, 9 kinds of physicochemical properties were selected in improved PseAAC [47]. The nine physicochemical properties are hydrophobicity, hydrophilicity, rigidity, flexibility, irreplaceability, side chain mass, pI at 25°C, pK of the *α*-COOH group, and pK of the *α*-NH_3_^+^ group [47], respectively. The original values of the physicochemical properties for 20 amino acids were all listed in Table 1. According to (2)–(7), each protein sample can be formulated by a (400 + 9*δ*) dimension vector including 400  *g*-gap dipeptide compositions and 9*δ* correlation factors based on physicochemical properties between two residues. From (3)–(5), the prediction performance of our method was influenced by two parameters, namely, *g* and *δ*, where *g* describes the local sequence-order effect and *δ* represents the global sequence-order effect. The current study searched for the optimal values for the two parameters according to the following standard:

(10) 0≤g≤9,with  step  Δ=11≤δ≤10,with  step  Δ=1.

In cross-validation test,* n*-fold cross-validation, jackknife cross-validation, and independent dataset test are often used for measuring the performance of prediction model. Although the jackknife cross-validation is deemed the most objective because it can always yield a unique result for benchmark dataset given [59, 60] and it has been more and more widely used, it also has obvious drawbacks, such as the large calculation and being time-consuming. Hence, the 5-fold cross-validation was adopted in this work for searching the optimal parameters and the optimal feature subset. Once the optimal feature subset was determined, we used jackknife cross-validation for verification ulteriorly.

**Figure 1:**
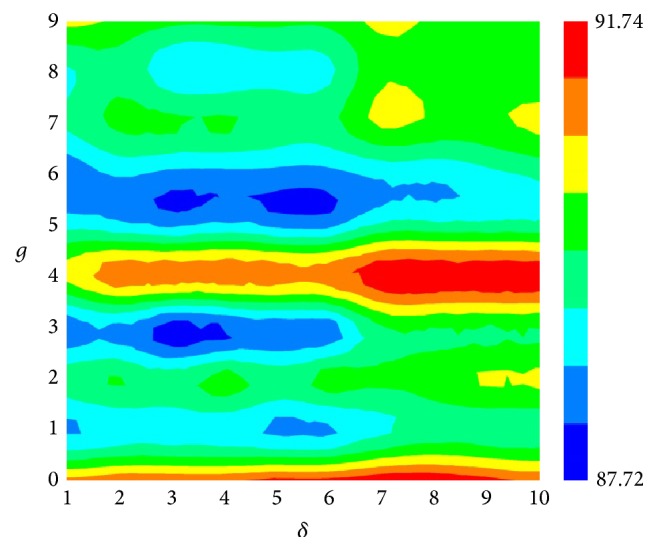
A heat map to show the overall accuracy in 5-fold cross-validation with different parameter groups (*g*, *δ*).

Based on (10), a total of 10 × 10 = 100 groups of parameters (*g*, *δ*) were investigated. For each parameter group (*g*, *δ*), there are 400 + 9*δ* feature subsets. Then, we used feature selection technique defined in (8) to find out the best one in each parameter group. Thus, we obtained the 100 highest OAs for 100 groups of parameters (*g*, *δ*). To provide an overall and intuitive analysis, the best OAs were drawn into a heat map, where the column and row of the heat map represent the parameters *g* and *δ*, respectively. Each element in the heat map represents one of the 100 groups of parameters (*g*, *δ*) and was colorized according to its highest overall accuracy in feature selection process. From Figure 1, we noticed that several elements are red indicating the maximum overall accuracy of 91.73% when *g* equals 0 or 4 and *δ* equals 7, 8, 9, and 10. Generally, a model with a small number of features can reduce the risk of overfitting. After checking the feature selected results, we found that when using feature selection technique to optimize parameter group (*g* = 4 and *δ* = 7), the optimal feature subset contains 63 features, which is less than the optimal feature subset in other groups. Thus, the final model was established based on the 63 features from parameter group (*g* = 4 and *δ* = 7).

**Figure 2:**
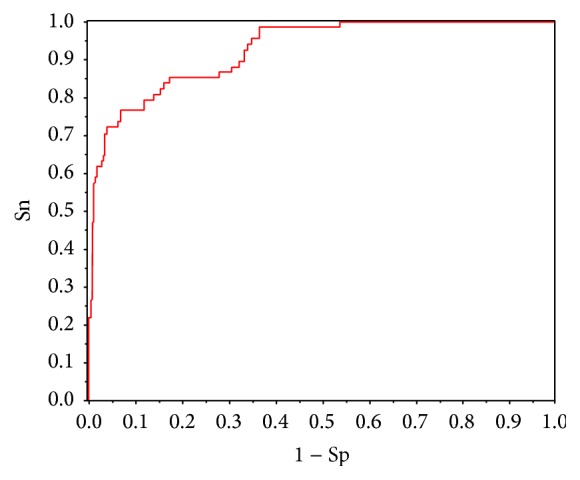
The ROC curve for the proposed model with the 63 optimal features in jackknife cross-validation using SVM.

Because there is imbalance in our benchmark dataset, the average accuracy and ROC curve were employed to evaluate the model. Thus, we set a series of different classification thresholds to seek the maximum of average accuracy. The maximum AA and corresponding Sn, Sp, MCC, and OA were listed in Table 2. The ROC curve can demonstrate the predictive capability of the proposed method across the entire range of SVM decision values. Thus, we plotted the ROC curve in Figure 2. It shows that auROC is 0.926, demonstrating that our model has capability to predict cell wall lyases.

To investigate whether other algorithms have the same or higher discriminate capability in the same feature space, the performances of Random Forest, Naïve Bayes, and LibD3C were examined by using jackknife cross-validation. Random Forest and Naïve Bayes were executed by using free package WEKA [61]. The LibD3C, a new selective ensemble algorithm, is a hybrid model of ensemble pruning that is based on *k*-means clustering and the framework of dynamic selection and circulating in combination with a sequential search method [30]. We used default parameters in LibD3C to perform classification.

The jackknife cross-validated results were also recorded in Table 2 for clear comparison. Note that the result for each algorithm in Table 2 was calculated with the maximum AA. As can be seen from the table, although Sn's of Random Forest and Naïve Bayes are no lower than SVM, other indicators (Sp, MCC, OA, AA, and auROC) of SVM are the best.

### 3.2. Online-Server Guide

A user-friendly online server called* Lypred* was established. A simple guide about the server was given below in order to further make it easier for the users.

**Figure 3:**
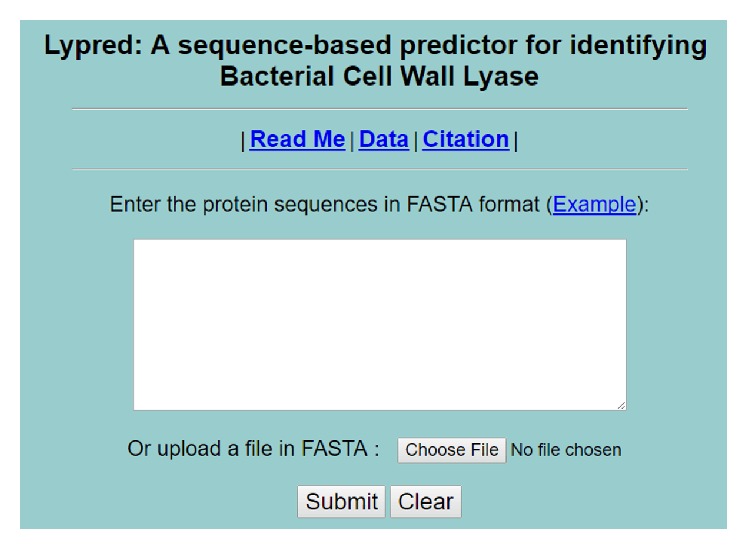
A semiscreenshot to show the home page of Lypred. Its website address is http://lin.uestc.edu.cn/server/Lypred/.

*Lypred* has five pages. Users can browse the server at http://lin.uestc.edu.cn/server/Lypred/ and see the home page on the screen as shown in Figure 3. The Read Me page provides a brief introduction about* Lypred* and the caveat when being used. The Data page shows a brief description about the benchmark dataset and the optimal feature subset used in this work and provides links for downloading. The relevant paper about the detailed development and algorithm of* Lypred* can be seen by clicking the Citation button. Example sequences in FASTA format can be found by clicking the Example button right above the input box.

Users can not only type or copy/paste the query protein sequences into the input box, but also upload FASTA/txt file containing the query protein sequences at the center of the home page of* Lypred*. Note that* Lypred* also has some constraints so as to guarantee the reliability of the results: firstly, protein sequences must be in FASTA format consisting of a single initial line beginning with a greater-than symbol (“>”) in the first column, followed by lines of sequence data, and the sequence is deemed to end if there is another line starting with “>”; secondly, the query protein sequence should only contain 20 kinds of amino acids; thirdly, the length of a query protein sequence should be no less than eight.

## 4. Conclusions

With growing drug resistance of pathogenic bacteria, great effort has been placed on the study and development of lyases. Effective identification of lyases will provide convenience for development of new antimicrobials. In this work, we used an improved PseAAC including *g*-gap dipeptide compositions and correlation factors of the physicochemical properties to extract the characteristics of protein sequences. A feature selection technique based on ANOVA was used to optimize features. The results of AA of 84.82% and auROC of 0.926 make us believe that* Lypred* will become a powerful and useful tool for the experimental study of bacterial cell wall lyase.

## Figures and Tables

**Table 1:** The original values of nine physicochemical properties used in this study.

Amino acids	Hydrophobicity	Hydrophilicity	Rigidity	Flexibility	Irreplaceability	Mass	pI	pK(*α*-COOH)	pK(*α*-NH_3_^+^)
A	0.62	−0.5	−1.338	−3.102	0.52	15	6.11	2.35	9.87
C	0.29	−1	−1.511	0.957	1.12	47	5.02	1.71	10.78
D	−0.9	3	−0.204	0.424	0.77	59	2.98	1.88	9.6
E	−0.74	3	−0.365	2.009	0.76	73	3.08	2.19	9.67
F	1.19	−2.5	2.877	−0.466	0.86	91	5.91	2.58	9.24
G	0.48	0	−1.097	−2.746	0.56	1	6.06	2.34	9.6
H	−0.4	−0.5	2.269	−0.223	0.94	82	7.64	1.78	8.97
I	1.38	−1.8	−1.741	0.424	0.65	57	6.04	2.32	9.76
K	−1.5	3	−1.822	3.950	0.81	73	9.47	2.2	8.9
L	1.06	−1.8	−1.741	0.424	0.58	57	6.04	2.36	9.6
M	0.64	−1.3	−1.741	2.484	1.25	75	5.74	2.28	9.21
N	−0.78	0.2	−0.204	0.424	0.79	58	10.76	2.18	9.09
P	0.12	0	1.979	−2.404	0.61	42	6.3	1.99	10.6
Q	−0.85	0.2	−0.365	2.009	0.86	72	5.65	2.17	9.13
R	−2.53	3	1.169	3.060	0.60	101	10.76	2.18	9.09
S	−0.18	0.3	−1.511	0.957	0.64	31	5.68	2.21	9.15
T	−0.05	−0.4	−1.641	−1.339	0.56	45	5.6	2.15	9.12
V	1.08	−1.5	−1.641	−1.339	0.54	43	6.02	2.29	9.74
W	0.81	−3.4	5.913	−1.000	1.82	130	5.88	2.38	9.39
Y	0.26	−2.3	2.714	−0.672	0.98	107	5.63	2.2	9.11

**Table 2:** Comparison among the performances of different algorithms.

Algorithm	Sn (%)	Sp (%)	MCC	OA (%)	AA (%)	auROC
SVM	76.47	93.16	0.678	90.13	84.82	0.926
Random Forest	80.88	85.02	0.572	84.27	82.95	0.905
Naïve Bayes	76.47	83.06	0.512	81.87	79.77	0.881
LibD3C	66.18	88.60	0.515	84.53	77.39	0.859
